# *VEGFA rs3025020* Polymorphism Contributes to *CALR* -Mutation Susceptibility and Is Associated with Low Risk of Deep Vein Thrombosis in Primary Myelofibrosis

**DOI:** 10.1055/s-0041-1739293

**Published:** 2021-11-09

**Authors:** Laura Villani, Vittorio Rosti, Margherita Massa, Rita Campanelli, Paolo Catarsi, Adriana Carolei, Carlotta Abbà, Annalisa de Silvstri, Robert Peter Gale, Giovanni Barosi

**Affiliations:** 1 Center for the Study of Myelofibrosis, Laboratory of Biochemistry, Biotechnology and Advanced Diagnostics, Istituto di Ricovero e Cura a Carattere Scientifico Policlinico S. Matteo Foundation, Pavia, Italy; 2 Laboratory of Biochemistry, Biotechnology and Advanced Diagnostics, Istituto di Ricovero e Cura a Carattere Scientifico Policlinico San Matteo Foundation, Pavia, Italy; 3 Biometry & Clinical Epidemiology, Scientific Direction, Istituto di Ricovero e Cura a Carattere Scientifico Policlinico San Matteo Foundation, Pavia, Italy; 4 Department of Immunology and Inflammation, Centre for Haematology Research, Imperial College London, London SW7 2BU, United Kingdom

**Keywords:** primary myelofibrosis, *VEGFA* polymorphism, *rs3025020*, deep vein thrombosis, *CALR* mutation

## Abstract

**Background** Single nucleotide polymorphisms (SNPs) in vascular endothelial growth factor A ( *VEGFA* ) are associated with susceptibility to several diseases including cancer. Correlations between *VEGFA rs3025020* genotypes with clinical and laboratory features of primary myelofibrosis (PMF) are unstudied.

**Methods** DNA was analyzed by real-time polymerase chain reaction for *VEGFA rs3025020* genotypes in a cohort of 844 subjects with PMF and in two cohorts of normal subjects ( *N* = 247 and *N* = 107).

**Results** Frequency of *rs3025020* minor allele (T) was not significantly different in subjects with PMF compared with normals; however, the T-allele was more frequent in PMF subjects with a calreticulin ( *CALR* )-mutated genotype compared with normals (35 vs. 27%; OR = 1.47 [95% CI, 1.09, 1.98] *p* = 0.011), especially in subjects with a *CALR-* type 2/type 2-like mutation (43 vs. 27%; OR = 2.01 [1.25, 3.24] *p* = 0.004). *CALR* mutants with the *rs3025020* TT genotype had higher CXCR4 expression on CD34-positive blood cells, and those who carried CT/TT genotypes had lower platelet concentrations compared with other genotypes at diagnosis. Overall, subjects with the *rs3025020* CT/TT genotype had a lower cumulative incidence of deep vein thrombosis in typical sites (1.6 vs. 4.2%; OR = 0.37 [0.15, 0.90] *p* = 0.029) and longer interval from diagnosis to first thrombosis (HR = 0.37 [0.14, 0.95] *p* = 0.039).

**Conclusion** Persons with PMF and the *VEGFA rs3025020* minor T-allele are more likely to have a *CALR* mutation compared with other somatic driver mutations and lower cumulative incidence and hazard for deep vein thrombosis in typical sites.

## Introduction

Primary myelofibrosis (PMF) is a myeloproliferative disorder mostly caused by g *ain-of-function* driver mutations in Janus kinase-2 ( *JAK2* ), calreticulin ( *CALR* ), or myeloproliferative leukemia virus ( *MPL* ). 1 Persons with these mutations often have additional variations in *ASXL1, EZH2, DMNT3A, IDH1* , and *IDH2* . 2 However, the mutation topography of PMF does not completely account for the different phenotypes including clinical and laboratory co-variates and risks of thrombosis, progression, and transformation to acute myeloid leukemia. Other gene loci are also important. For example, considerable data indicate some single nucleotide polymorphisms (SNPs) such as *JAK2* , *MECOM* , *TERT* , *HBS1L-MYB* , *THRB-RARB* , glucocorticoid receptor, and monocyte chemoattractant protein-1 predispose to developing PMF and/or correlate with clinical and prognostic features. 3 4 5 6 7 8 9 10 11

Vascular endothelial growth factor A (VEGFA) is a pro-angiogenic protein correlated with the development and progression of myeloproliferative neoplasms, including PMF. 12 SNPs in *VEGFA* are associated with susceptibility to several diseases, including cancer. 13 14 15 We now report correlations between *VEGFA rs3025020* genotypes and clinical and laboratory features of PMF.

## Materials and Methods

### Study Population

Stored DNA from blood granulocytes of 844 consecutive subjects with PMF, seen at the Center for the Study of Myelofibrosis of the IRCCS Policlinico S. Matteo Foundation in Pavia and included in a database, was the primary source material of this study. Clinical data were collected on the first visit and prospectively thereafter. Diagnosis was confirmed by reviewing the initial bone marrow biopsy and based on the WHO diagnostic criteria at the time of their first visit and re-classified according to 2017 revised WHO criteria. 16 After the first examination, visits were scheduled every 6 months.

### Control Populations

We used two control cohorts, one of healthy Italian participants in a bone marrow donor registry whose blood samples were anonymized ( *N* = 247), and data generated from the public database 1000 Genomes Project_Phase 3_EUR_Subpopulation (Tuscans in Italy) ( *N* = 107).

### SNP Analysis

DNA was isolated from blood granulocytes, obtained by density gradient centrifugation, using the QIAamp DNA Blood Mini Kit (QIAGENSciences Inc. Germantown, Maryland, United States). SNP genotyping used a pre-designed, two-labeled (VIC-FAM) TaqMan Assay C_1647366_10 (Applied Biosystems, Foster City, California, United States). Reactions were done on a CFX96 Realtime PCR Detection System (Biorad Company, Hercules, California, United States) according to the manufacturer's instructions.

### Data Analyzed

Data were collected at diagnosis and analyzed included age, spleen size by clinical measurement, CBC with differential, serum lactate dehydrogenase level and cholesterol concentration, percentage of blood blasts, and International Prognostic Scoring System (IPSS) risk category. 17 In most subjects, blood concentrations of CD34-positive cells, 18 and blood CXCR4 expression on CD34-positive cells were also quantified. 19 Diagnoses of bone marrow biopsies were analyzed and fibrosis graded according to EUMNET consensus criteria. 20 Cytogenetic analyses used standard techniques and were reported according to the International System for Human Cytogenetic Nomenclature criteria. 21

*JAK2 ^V^*^617F^ and *MPL* ^W515^ were detected by the real-time polymerase chain reaction or high-resolution melting analyses. *CALR* mutations were identified by capillary electrophoresis and bi-directional sequencing. Next generation sequencing was used to detect mutations in selected myeloid neoplasm-related genes. Genomic and transcript analyses were performed using the diagnostic panel commercially available Oncomine Myeloid Research Assay (Thermofisher, Waltham, Massachusetts, United States). The genomic and transcript analysis were performed with IonReporter software applying the last release of Myeloid workflow (Thermofisher). Variations causing missense, frameshift, an altered stop/initiation codon, in-frame insertion/deletion or variants affecting splice site were regarded as mutations.

### Statistical Analyses

Analyses considered clinical and laboratory co-variates at diagnosis. Continuous variables were presented as means (+SD) or median with interquartile range (IQR). Categorical variables were expressed as percentages. Pearson *χ* -test with one degree of freedom was used to compare allele and genotypes frequencies. Deviations from the Hardy-Weinberg equilibrium were tested by the Fisher exact test.

The independent contribution of *rs3025020* SNP to odds of PMF phenotypes was assessed by logistic regression analysis. Since this was an exploratory study, we did not introduce a correction for multiple comparisons. 22 End points for associations with genotypes were blast transformation-free survival and survival. We further analyzed the outcome of major thromboses, including arterial thromboses (myocardial infarction, stroke), deep vein thrombosis and pulmonary embolism, and thrombosis in atypical sites (portal vein thrombosis, Budd-Chiari syndrome, and cerebral sinus thrombosis). Competing risk analysis was used to compute thrombosis cumulative incidence rates, considering overall mortality as the competing event. The Fine and Gray competing risks regression was used to study the determinants of thromboses. 23 Subhazard ratio (sHR) and 95% CI were computed. In this analysis, conventional risk factors measured at diagnosis, including age, sex, *JAK2* ^V617F^ mutation, *CALR* mutation, white-blood cell count were analyzed. In multivariable regression, non-collinear variables associated with thrombosis with *p* < 0.1 at univariable analysis, and with a frequency of missing values lower than 20%, were used to assess whether *VEGFA rs3025020* status independently predicted thrombotic occurrence. Results were considered statistically significant when two-sided *p* -values were less than 0.05. These computations were performed with STATA 12 (Stata Corporation, College Station, Texas, United States).

All other computations were performed with STATISTICA software **(** Dell Technologies Inc. Round Rock, Texas, United States).

## Results

Demographic and clinical co-variates of the 844 subjects with PMF are displayed in Table 1 . In total, 499 were male (59%). Median age was 52 years (IQR, 40–61). Fifty-seven percent had a prefibrotic myelofibrosis. The IPSS risk distribution was low in 61%, intermediate-1 in 17%, intermediate-2 in 14%, and high in 9% of the subjects. *JAK2* ^V617F^ was detected in 66% of subjects tested, 60% of whom had a heterozygous mutation, and 40% had a homozygous mutation. Thirty-nine of 136 subjects tested by cytogenetics (29%) had an abnormal karyotype. A total of 72 subjects (14%) had died after a median follow-up of 77 months (IQR, 36–150). Median survival is 21 years. Eighty-eight subjects (17%) had blast transformation (BT) at a median of 26 years.

**Table 1:** Baseline characteristics of subjects with primary myelofibrosis at diagnosis analyzed for *rs3025020 VEGFA* polymorphism

	Primary myelofibrosis ( *N* = 844)
Demography	
Age, y, median (IQR)	52 (40–61)
Sex male, *N* (%)	499 (59.1)
Clinical-hematological co-variates	
Hemoglobin, g/L, mean (±SD)	128 (29)
White blood cell count × 10E + 9/L, mean (±SD)	9.9 (6.4)
Platelet count × 10E + 9/L, mean (±SD)	510 (347)
Monocyte count × 10E + 9/L, mean (±SD)	606 (507)
Spleen size, cm E + 2, mean (±SD)	148 (99)
IPSS score, low, *N* (%)	515 (61)
IPSS score, intermediate-1, *N* (%)	144 (17)
IPSS score, intermediate-2, *N* (%)	110 (13)
IPSS score, high, *N* (%)	77 (9)
Plasma LDH, x ULN, mean (±SD)	1.64 (1.13)
Serum cholesterol, mg/dL, mean (±SD)	159 (43)
Blood CD34-positive cells × 10E + 6/L, mean (±SD)	59 (160)
CXCR4 expression on blood CD34-positive cells, %, mean (±SD)	42 (25)
Molecular characteristics	
* JAK2*^V617F^*N* (%)	541 (65.7)
* CALR* mutation, *N* (%)	171 (20.7)
* MPL* mutation, *N* (%)	44 (53.5)
* Triple negative* , *N* (%)	67 (8.1)
* ASXL1* or *EZH2* mutations,	
• positive, *N* (%)	45 (18.7)
• negative, *N* (%)	195 (81.3)
Bone marrow histology (fibrosis)	
Grade-0, *N* (%)	259 (30.8)
Grade-1, *N* (%)	225 (26.7)
Grade-2, *N* (%)	241 (28.6)
Grade-3, *N* (%)	117 (13.9)

Abbreviations: IQR, interquartile range; LDH, lactic dehydrogenase; SD, standard deviation; *Triple negative* , subjects without any of the myeloproliferative neoplasm driver mutations ( *JAK2* ^V617F^ , *CALR* , *MPL* ^W515^ ); ULN, upper limit of normal.

Note: Spleen size was measured using the spleen index calculated by multiplying the length of the longitudinal axis by the transverse axis. Monocyte concentration was available in 452 subjects; plasma LDH activity was available in 469 subjects; serum cholesterol concentration was available in 421 subjects; blood CD34-positive cell concentration was available in 398 subjects; CXCR4 expression on CD34-positive blood cells was available in 295 subjects.

### Association with *VEGFA rs3025020*

Genotype frequency distributions of *VEGFA rs3025020* were consistent with the Hardy-Weinberg equilibrium in subjects with PMF and controls. Frequencies of the *VEGFA rs3025020* alleles in PMF subjects did not differ significantly from local controls (OR = 1.19 [0.95, 1.40] *p* = 0.11), and “Tuscans in Italy” cohort (OR = 1.02 [0.75, 1.39] *p* = 0.89; Table 2 ).

**Table 2:** Genotype and allele frequencies of the *rs3025020* polymorphism of *VEGFA* in 844 patients with primary myelofibrosis (PMF), 247 subjects of healthy control population, and 107 subjects reported from “Tuscan from Italy”

	*N*	*rs3025020* polymorphism					
		CC	CT	TT	CC/CT	CT/TT	T allele frequency
PMF subjects, *N* (%)	844	407 (48.2)	354 (41,9)	83 (9.8)	761 (90.2)	437 (51.8)	520/1688 (30.8)
Local healthy controls, *N* (%)	247	134 (54.3)	92 (37.2)	21 (8.5)	226 (91.4)	113 (45.7)	134/494 (27.1)
Tuscans from Italy, *N* (%)	107	51 (47.7)	47 (43.9)	9 (8.4)	98 (91.6)	56 (52.3)	65/214 (30)

### *VEGFA rs3025020* SNP and PMF Somatic Driver Mutations

PMF cohorts defined by the somatic driver mutations, i.e., *JAK2* ^V617F^ , *CALR, MPL* mutants, or the so-called “triple negative” (no detectable driver mutation) were in Hardy-Weinberg equilibrium ( Supplementary Table S1 ). Compared with local healthy controls, *rs3025020* T-allele variant was not enriched in subjects with *JAK2* ^V617F^ (28.8 vs. 27.1%; OR = 1.0 [0.85, 1.37] *p* = 0.49). Conversely, in subjects without *JAK2* ^V617F^ the *VEGFA rs3025020* T-allele was more frequent than in the local controls (34 vs. 27.1%; OR, 1.39 [1.04, 1.80] *p* = 0.015), and was higher than subjects with *JAK2* ^V617F^ (37.2 vs. 28.8%; OR = 1.27 [1.02, 1.58] *p* = 0.028).

Subjects with a *CALR* mutation had a higher *VEGFA rs3025020* T-allele frequency compared with local controls (35.4 vs. 27.1%; OR = 1.47 [1.09, 1.98] *p* = 0.011), mostly because of an increased frequency in subjects with a *CALR-* type 2/type 2-like mutation compared with controls (42.8 vs. 27.1%; OR = 2.01 [1.25, 3.24] *p* = 0.004).

### *VEGFA rs3025020* Genotype and PMF Co-variates

Associations between *VEGFA rs3025020* genotypes and PMF co-variates at diagnosis are displayed in Supplementary Table S2 . There were no significant correlations with age or sex. Subjects with the CT/TT genotype had a higher mean white blood cell (WBC) count compared with the CC genotype (10.4 vs. 9.5 × 10E + 9/L; *p* = 0.04). Subjects with the *VEGFA rs3025020* TT genotype had higher mean CXCR4 expression on CD34-positive cells compared with those with the CC/CT genotypes (54.4 vs. 40.8%; *p* = 0.005). We detected no significant association between *rs3025020* genotype and frequency of non-driver clonal mutations, cytogenetic abnormalities, or degree of bone marrow fibrosis at diagnosis.

We found inconsistent correlations between WBC or CXCR4 expression on CD34-positive cells and driver mutations in the whole cohort of PMF subjects. The association between *VEGFA rs3025020* CT/TT genotype and higher mean WBC count was statistically significant only in *JAK2* ^V617F^ cohorts (11.5 vs. 9.8 × 10E + 9/L; *p* = 0.005; Supplementary Table S3 ), and was only significant in subjects with an allele burden >50% (homozygous genotype) (14.7 vs. 11.3 × 10E9/L; *p* = 0.002) ( Supplementary Table S4 ). Higher CXCR4 expression on CD34-positive cells was significant in subjects with the *rs3025020* TT genotype compared with the CT/CC genotype only in subjects without *JAK2* ^V617F^ (59.9 vs. 23.7%; *p* = 0.002; Supplementary Table S5 ), and this association was detected only in subjects with a *CALR* mutation ( Supplementary Tables S6–S10 >).

We also detected an association between the *VEGFA rs3025020* CT/TT genotype and a lower platelet concentration compared with the CC genotype in subjects with a *CALR* mutation (mean: 629 vs. 782 × 10E + 9/L; *p* = 0.013; Supplementary Table S7 ). Associations of the *VEGFA rs3025020* CT/TT genotype with a lower platelet concentration at diagnosis and the *rs3025020* TT genotype with higher CXCR4 expression on CD34-positive cells were detected in type 1/type 1-like and type 2/type 2-like *CALR* mutation but were statistically significant in type 2/type 2-like *CALR* mutation (993 vs. 617 × 10E +/L; *p* = 0.0031; and 69 vs. 32%; *p* < 0.001; Supplementary Table S8 ).

### *VEGFA rs3025020* Genotype and Thrombosis

In total, 168 subjects (20%) had a major thrombotic event. Cumulative frequency of the first thrombotic event was 8.7, 10.4 and 15.9% in CARL- and MPL-mutated and triple negative individuals, respectively. On the contrary, subjects carrying the *JAK2* ^V617F^ mutation had the highest risk of thrombosis compared with all the other groups (25.2%). 56.5% of the subjects had vein thrombosis in atypical sites, 77% of which were synchronous with PMF diagnosis ( Table 3 ). Twenty-nine percent of the subjects had arterial thrombosis and 14.3% had deep vein thrombosis in typical sites. The frequency of thrombotic events was lower in subjects with the *VEGFA rs3025020* CT/TT genotypes compared with those with the *VEGFA rs3025020* CC genotype (17.8 vs. 22.1%; OR, 0.76 [0.54, 1.07] *p* = 0.117). A lower frequency of thrombotic events in subjects with the *VEGFA rs3025020* CT/TT genotype was detected in the three thrombosis categories but significant only in the deep vein thromboses in typical site category (OR = 0.37 [0.15, 0.90] *p* = 0.029). Subjects with the *rs3025020* TT or CT genotype ( *N* = 437) developed seven events (1.6%), with a post-diagnosis cumulative event rate of 0.21 *per* 100 person-years compared with a *VEGFA rs3025020* CC genotype ( *N* = 406) who had 17 events (4.2%) with a post-diagnosis cumulative event rate of 0.60 *per* 100 person–years ( *p* = 0.025).

**Table 3:** Major thrombotic events stratified for *rs3025020 VEGFA* polymorphism

	*rs3025020* polymorphism						CC vs. CT/TT Odds ratio (95% CI)	TT vs. CC/CT Odds ratio (95% CI)
	All cases	CC	CT	TT	CC/CT	CT/TT		
	843	406	354	83	760	437		
Overall thrombotic events, *N* (% of PMF cases)	168 (19.9)	90 (22.1)	66 (18.6)	12 (14.4)	156 (20.5)	78 (17.8)	0.76 (0.54, 1.07) *p* = 0.11	0.65 (0.34, 1.23) *p* = 0.19
Arterial thrombosis, *N* (% of PMF cases)	49 (5.8)	28 (6.9)	17 (4.8)	4 (4.8)	45 (5.9)	21 (4.9	0.68 (0.38,1.22) *p* = 0.19	0.80 (0.28, 2.29) *p* = 0.68
• In year before diagnosis, *N* (% of thromboses)	14 (28.6)	7 (25)	7 (41.2)	0 (0)	14 (31.1)	7 (33.3)		
• At diagnosis, *N* (% of thromboses)	12 (24.5)	9 (32.1)	2 (11.8)	1 (25)	11 (24.4)	3 (14.3)		
• After diagnosis, *N* (% of thromboses)	23 (46.9)	12 (42.8)	8 (47.1)	3 (75)	20 (44.4)	11 (52.4)		
Deep vein thrombosis in typical sites, *N* (% of PMF cases)	24 (2.8)	17 (4.2)	7 (1.9)	0 (0)	24 (3.1)	7 (1.6)	0.37 (0.15, 0.91) *p* = 0,029	0.18 (0.01, 2.98) *p* = 0.23
• In year before diagnosis, *N* (% of thromboses)	3 (12.5)	2 (11.8)	1 (14.3)	0 (0)	3 (12.5)	1 (14.3)		
• At diagnosis, *N* (% of thromboses)	5 (28.8)	3 (17.6)	2 (28.6)	0 (0)	5 (20.8)	2 (28.6)		
• After diagnosis, *N* (% of thromboses)	16 (66.6)	12 (70.6)	4 (23.5)	0 (0)	16 (66.6)	4 (57.1)		
Venous thrombosis in atypical sites, *N* (% of PMF cases)	95 (11.3)	45 (11.1)	42 (11.9)	8 (9.6)	87 (11.4)	50 (11.4)	1.03 (0.67, 1.59) *p* = 0.86	1.76 (0.79, 3.93) *p* = 0.16
• In year before diagnosis, *N* (% of thromboses)	7 (7.4)	1 (2.2)	5 (11.9)	1 (12.5)	6 (6.9)	6 (12)		
• At diagnosis, *N* (% of thromboses)	73 (76.8)	37 (82.2)	29 (69)	7 (87.5)	66 (75.9)	36 (72)		
• After diagnosis, *N* (% of thromboses)	15 (15.8)	7 (15.5)	8 (19)	0 (0)	15 (17.2)	8 (16)		

Abbreviation: PMF, primary myelofibrosis.

**Fig. 1:**
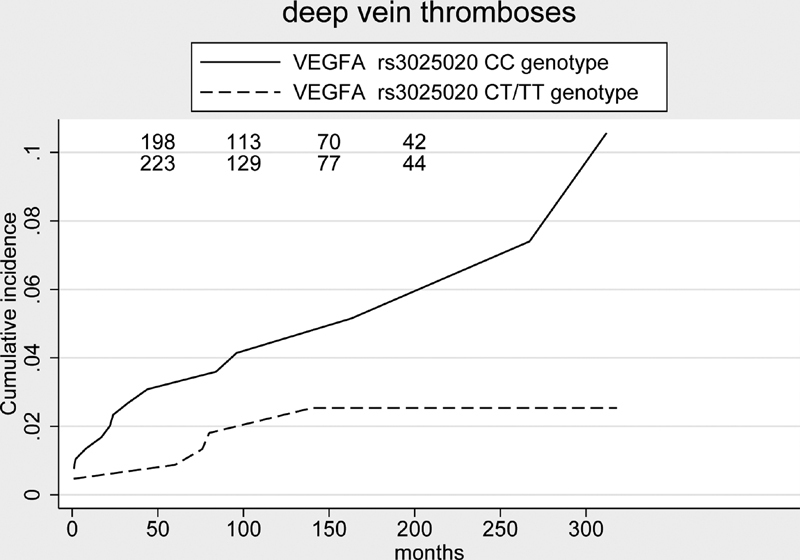
Cumulative incidence of deep vein thrombosis in typical sites in 844 subjects with PMF stratified for the *rs3025020 VEGFA* polymorphism genotypes: subjects with CT/TT genotype had a significant lower risk of thrombosis ( *p* = 0.039).

With the KM analysis, the *VEGFA rs3025020* genotype was significantly associated with the risk of incurring into deep vein thromboses in typical sites; it resulted higher in patients who had CC genotype with respect to those with CT/TT genotype (HR = 0.37 [0.14, 0.95] *p* = 0.038). With the Fine and Gray model, with thrombotic event of interest and overall mortality of the competing risk, the cumulative incidence of deep vein thrombosis in typical sites at the end of follow-up was 6.7% (95% CI, 3.1, 12.2) ( Fig. 1 ). When refining the assessment of *VEGFA rs3025020* status for the risk of thrombosis, we showed an increase in risk for CC genotype with respect to CT/TT genotype, with sHR for CT/TT 0.37 (0.14, 0.95) *p* = 0.039. At the time of thrombosis, 17% of patients were under cytoreductive therapy (90% hydroxyurea). Cytoreductive therapy use was not a prognostic factor of longer thrombosis free survival in the Cox proportional hazards model. When the sHR of thrombotic events was adjusted for the risk factors for thrombosis, the outcome resulted independently predicted by the *VEGFA rs3025020* CC genotype and by older age at the initial assessment ( Table 4 ).

**Table 4:** Multivariate proportional hazards regression: predictive factors for deep vein thrombosis in typical sites

Parameter	Hazard ratio	95% confidence interval	*p* -Value
Age at diagnosis >65 y	3.08	1.21, 7.82	0.018
*rs3025020* CT/TT	0.38	0.15, 0.96	0.042
*CALR* -mutated	0.55	0.13, 2.28	0.411
WBC count >12 × 10E + 9/L	1.31	0.51, 3.41	0.577
*JAK2*^V617F^ -positive	0.86	0.29, 2.52	0.782
Sex, male	0.75	0.30, 1.86	0.537

Abbreviations: CALR, calreticulin; WBC, white blood cells.

### *VEGFA rs3025020* Genotypes and Survival

We detected no significant correlations between *VEGFA rs3025020* genotypes and survival or risk of BT (HR, 1.02 [0.78, 1.34] *p* = 0.88; HR = 1.14 [0.82, 1.56] *p* = 0.42).

## Discussion

Our study indicates that persons with PMF with a *CALR* mutation, especially type 2/type 2-like, have an increased frequency of T-allele *VEGFA rs3025020* genotypes. This association is in keeping with data of other SNPs in persons with MPNs. Trifa et al reported associations of *TERT rs2736100* and *MECOM rs2201862* genotypes with *CALR* -mutated MPNs. 7 The *TERT rs2736100* correlation was independent of molecular subtype, whereas the *MECOM rs2201862* T-allele genotype was restricted to patients with type-1/type-1-like mutations. Lighezan et al reported associations between *TET2 rs1548483* genotype and *JAK2* ^V617F^ - and *CALR* -mutated PMF, especially with *CALR-* type 2 mutations. 24

The imbalance of *rs3025020* frequency in *CALR* -mutated PMF may arise directly from an effect(s) of the *VEGFA* polymorphism or linkage to other gene(s). We found subjects with a *CALR* mutation with the *VEGFA rs3025020* T-allele genotype having lower platelet concentrations at diagnosis than other *VEGFA rs3025020* genotypes, and those with the TT genotype having higher CXCR4 expression on CD34-positive cells compared with other genotypes. These correlations were strongest in subjects with *CALR-* type 2/type 2-like mutations.

In *CALR* -mutated PMF persons, thrombocytosis is the dominant phenotype reflecting myeloproliferation; in PMF, reduced CXCR4 expression on CD34-positive cells is associated with disease activity and poor prognosis. 19 This implies that the *VEGFA* r *s3025020* T-allele acts directly, or through tight linkage to another gene(s), to regulate myeloproliferation/disease activity in this setting. The correlations we found in *CALR* -mutated persons were poorly consistent across other driver mutations: in persons with homozygous *JAK2* ^V617F^ mutations we found association of the CT/TT genotype with a higher WBC concentration at diagnosis. The mechanism(s) underlying this requires further study.

The most interesting and clinically-important correlation we detected was the association between *rs3025020* and thrombosis risk. The high incidence of thrombosis in our subjects allowed us to distinguish between arterial and deep vein thromboses in typical and atypical sites. Subjects with a *rs3025020* T-allele genotype had a lower cumulative incidence and hazard of thromboses, particularly deep vein thrombosis in typical sites. Current knowledge about the potential impact of the *VEGFA rs3025020* on angiogenesis and endothelial function is limited and the mechanism(s) unknown.

The low frequency of deep vein thrombosis in our series did not allow to dissect whether the different PMF driver clonal mutations had influence on the risk of thrombosis. However, the coincidence of high susceptibility to *CALR* mutation and low risk of thrombosis in subjects bearing the T-allele of *rs3025020 VEGFA* polymorphism claims for a hypothetical explanation of the low risk of thrombosis in *CALR* -mutated PMF reported in literature 25 (we confirmed in this study).

Our study has limitations. First, it was retrospective, limiting the validity of interpreting some results, especially comparisons of rates and hazards of thromboses. Second, not adjusting for multiple comparison might result in false positives. However, the results of this study clearly illustrate and prove the concept that the constitutional genetic background is an important determinant of the risk of thrombosis in PMF.

In conclusion, we found that persons with PMF and *VEGFA rs3025020* minor T-allele genotypes are more likely to have a *CALR* driver mutation compared with other driver mutations and a lower cumulative incidence and hazard for deep vein thrombosis in typical sites. If validated there may be clinical and therapy implications.

## Notes

**What Is Known on This Topic?**

- Vascular endothelial growth factor A (VEGFA) is a special type of cytokine, which is secreted by various cells such as vascular endothelial cells, and plays an important role in regulating blood vessel formation, tumor growth and development and atherosclerosis. VEGFA is known to exert a major influence on the development and progression of primary myelofibrosis (PMF).
- The human *VEGFA* gene is located on chromosome 6p21.3, whose single nucleotide polymorphisms (SNPs) can affect gene expression by altering key regulatory sequences or by altering mRNA stability at key regulatory loci.
- *rs3025020 VEGFA* gene polymorphism is a predisposing polymorphism for many diseases including malignancy. No documentation of increased susceptibility to vascular disease has been reported. Correlations between *VEGFA rs3025020* genotypes with clinical and laboratory features of PMF are unstudied.

**What Does This Paper Add?**

- Subjects with PMF who carry the minor T-allele of the *rs3025020 VEGFA* polymorphism emerged as enriched in the *CALR* -mutated PMF.
- *CALR* -mutated subjects who carry *VEGFA rs3025020* CT/TT genotypes displayed lower platelet count and those carrying TT genotype displayed higher expression of CXCR4 on CD34-positive cells, both markers of myeloproliferation/disease activity.
- Individuals who carry the T-allele of *VEGFA rs3025020* had an overall lower risk of deep vein thrombosis in typical sites.
- Higher susceptibility of the *VEGFA rs3025020* T-allele in *CALR* -mutated PMF and lower risk of thrombosis in individuals carrying the T-allele provide a hypothetical explanation of the known low-thrombotic risk in *CALR* -mutated PMF.

## References

[1] Rumi E Trotti C Vanni D Casetti I C Pietra D Sant'Antonio E The genetic basis of primary myelofibrosis and its clinical relevance Int J Mol Sci 2020 21 23 8885 10.3390/ijms21238885 PMC7727658 33255170

[2] Grinfeld J Nangalia J Baxter E J Classification and personalized prognosis in myeloproliferative neoplasms N Engl J Med 2018 379 15 1416 1430 30304655 10.1056/NEJMoa1716614 PMC7030948

[3] Jones A V Chase A Silver R T JAK2 haplotype is a major risk factor for the development of myeloproliferative neoplasms Nat Genet 2009 41 04 446 449 19287382 10.1038/ng.334 PMC4120192

[4] Olcaydu D Harutyunyan A Jäger R A common JAK2 haplotype confers susceptibility to myeloproliferative neoplasms Nat Genet 2009 41 04 450 454 19287385 10.1038/ng.341

[5] Kilpivaara O Mukherjee S Schram A M A germline JAK2 SNP is associated with predisposition to the development of JAK2(V617F)-positive myeloproliferative neoplasms Nat Genet 2009 41 04 455 459 19287384 10.1038/ng.342 PMC3676425

[6] Tapper W Jones A V Kralovics R Genetic variation at MECOM, TERT, JAK2 and HBS1L-MYB predisposes to myeloproliferative neoplasms Nat Commun 2015 6 6691 25849990 10.1038/ncomms7691 PMC4396373

[7] Trifa A P Bănescu C Bojan A S MECOM, HBS1L-MYB, THRB-RARB, JAK2, and TERT polymorphisms defining the genetic predisposition to myeloproliferative neoplasms: a study on 939 patients Am J Hematol 2018 93 01 100 106 29047144 10.1002/ajh.24946

[8] Lighezan D L Bojan A S Iancu M *TET2* rs1548483 SNP associating with susceptibility to molecularly annotated polycythemia vera and primary myelofibrosis J Pers Med 2020 10 04 259 10.3390/jpm10040259 PMC7711989 33271790

[9] Tefferi A Lasho T L Mudireddy M The germline JAK2 GGCC (46/1) haplotype and survival among 414 molecularly-annotated patients with primary myelofibrosis Am J Hematol 2019 94 03 299 305 30516848 10.1002/ajh.25349

[10] Poletto V Rosti V Villani L A3669G polymorphism of glucocorticoid receptor is a susceptibility allele for primary myelofibrosis and contributes to phenotypic diversity and blast transformation Blood 2012 120 15 3112 3117 22879541 10.1182/blood-2012-05-433466 PMC3628115

[11] Masselli E Carubbi C Cambò B The -2518 A/G polymorphism of the monocyte chemoattractant protein-1 as a candidate genetic predisposition factor for secondary myelofibrosis and biomarker of disease severity Leukemia 2018 32 10 2266 2270 29568096 10.1038/s41375-018-0088-y PMC6170394

[12] Gadomska G Stankowska K Boinska J VEGF-A, sVEGFR-1, and sVEGFR-2 in BCR-ABL negative myeloproliferative neoplasms Medicina (Kaunas) 2017 53 01 34 39 28237691 10.1016/j.medici.2017.01.004

[13] Ferrara N Pathways mediating VEGF-independent tumor angiogenesis Cytokine Growth Factor Rev 2010 21 01 21 26 20005148 10.1016/j.cytogfr.2009.11.003

[14] Metzger C S Koutsimpelas D Brieger J Transcriptional regulation of the VEGF gene in dependence of individual genomic variations Cytokine 2015 76 02 519 526 26209503 10.1016/j.cyto.2015.07.015

[15] Eng L Azad A K Habbous S Vascular endothelial growth factor pathway polymorphisms as prognostic and pharmacogenetic factors in cancer: a systematic review and meta-analysis Clin Cancer Res 2012 18 17 4526 4537 22733538 10.1158/1078-0432.CCR-12-1315

[16] Swerdow S H Campo E Harris N L WHO Classification of Tumours of Haematopoietic and Lymphoid TissuesRevised 4th ed.IARC Lyon 2017

[17] Cervantes F Dupriez B Pereira A New prognostic scoring system for primary myelofibrosis based on a study of the International Working Group for Myelofibrosis Research and Treatment Blood 2009 113 13 2895 2901 18988864 10.1182/blood-2008-07-170449

[18] Barosi G Viarengo G Pecci A Diagnostic and clinical relevance of the number of circulating CD34(+) cells in myelofibrosis with myeloid metaplasia Blood 2001 98 12 3249 3255 11719361 10.1182/blood.v98.12.3249

[19] Barosi G Rosti V Catarsi P Reduced CXCR4-expression on CD34-positive blood cells predicts outcomes of persons with primary myelofibrosis Leukemia 2021 35 02 468 475 32536689 10.1038/s41375-020-0926-6

[20] Thiele J Kvasnicka H M Myelofibrosis—what's in a name? Consensus on definition and EUMNET grading Pathobiology 2007 74 02 89 96 17587880 10.1159/000101708

[21] Larson D P Akkari Y M Van Dyke D L Conventional cytogenetic analysis of hematologic neoplasms: a 20-year review of proficiency test results from the College of American Pathologists/American College of Medical Genetics and Genomics Cytogenetics Committee Arch Pathol Lab Med 2021 145 02 176 190 32886736 10.5858/arpa.2020-0089-CP

[22] Streiner D L Norman G R Correction for multiple testing: is there a resolution? Chest 2011 140 01 16 18 21729890 10.1378/chest.11-0523

[23] Fine J P Gray R J A proportional hazards model for the subdistribution of a competing risk J Am Stat Assoc 1999 94 496 509

[24] Trifa A P Bănescu C Tevet M TERT rs2736100 A>C SNP and JAK2 46/1 haplotype significantly contribute to the occurrence of JAK2 V617F and CALR mutated myeloproliferative neoplasms—a multicentric study on 529 patients Br J Haematol 2016 174 02 218 226 27061303 10.1111/bjh.14041

[25] Finazzi M C Carobbio A Cervantes F CALR mutation, MPL mutation and triple negativity identify patients with the lowest vascular risk in primary myelofibrosis Leukemia 2015 29 05 1209 1210 25482134 10.1038/leu.2014.343

