# Supplementary material for: VEGFA rs3025020 Polymorphism Contributes to CALR -Mutation Susceptibility and Is Associated with Low Risk of Deep Vein Thrombosis in Primary Myelofibrosis
Source: TH Open. 2021 Nov 9;5(4):e513–20. doi: 10.1055/s-0041-1739293 (PMC8577885; doi:10.1055/s-0041-1739293)
Supplement: Supplementary file 1 — Supplementary Material [file 10-1055-s-0041-1739293-s210038.pdf]

Supplementary Table S1 Genotype and allele frequencies of the *rs3025020 VEGFA* variant in subjects with PMF stratified according to the categories defined by the PMF somatic driver mutations

	Number of cases	CC genotype	CT genotype	TT genotype	CC/CT genotype	CT/TT genotype	T-allele frequency	vs. local healthy controls, OR (95% CI); *P*[a]
JAK2^{V617F} positive								
N (%)	540	275 (50.9)	219 (40.5)	46 (8.5)	494 (91.5)	265 (49.1)	311/1,080 = 28.8%	**OR = 1.08 (0.85, 1.37); *p* = 0.49**
JAK2^{V617F} heterozygous								
N (%)	336	179 (53.3)	132 (39.3)	25 (7.4)	311 (92.5)	157 (46.7)	182/672 = 27.1%	**OR = 0.99 (0.76, 1.29); *p* = 0.98**
JAK2^{V617F} homozygous								
N (%)	204	96 (47)	87 (42.6)	21 (10.3)	183 (89.7)	108 (52.9)	129/408 = 31.6%	**OR =1.24 (0.93, 1.65); *p* = 0.14**
JAK2^{V617F} negative								
N (%)	282	124 (51.2)	124 (51.2)	34 (12.1)	248 (87.9)	158 (56)	192/564 = 34%	**OR = 1.39 (1.04, 1,80): *p* = 0.015**
CALR-positive								
N (%)	171	71 (41.5)	79 (46.2)	21 (12.3)	150 (87.7)	100 (58.5)	121/342 = 35.4%	**OR = 1.47 (1.09, 1.98): *p* = 0.011**
CALR-type 1								
N (%)	118	52 (44.1)	56 (47.5)	10 (8.5)	108 (91.5)	66 (55.9)	76/236 = 32.2%	**OR =1.27 (0.91, 1.78); *p* = 0.16**
CALR-type 2								
N (%)	42	14 (33.3)	20 (47.6)	8 (19)	34 (80.9)	28 (66)	**36/84 = 42.8%**	**OR = 2.01 (1.25, 3.24); *p* = 0.004**
MPL positive								
N (%)	44	24 (54.5)	11 (25)	9 (11.2)	35 (79.5)	20 (45.4)	29/88 = 32.9%	OR = 1.32 (0.81, 2.14): *p* = 0.26
Triple negative[b]								
N (%)	67	29 (43.3)	34 (50.7)	4 (6)	63 (94)	38 (56.7)	42/134: 31.3%	OR = 1.22 (0.81, 1.86);*p* = 0.33

[a]T allele frequency of local healthy controls: 134/494 = 27.1%

[b]*Triple negative* were subjects without any of the myeloproliferative neoplasm driver mutations (*JAK2*^{V617F}, *CALR*, *MPL*^{W515}).

 © 2021. The Author(s).

Supplementary Table S2 Clinical and laboratory features at diagnosis of the whole cohort of patients with PMF stratified by the genotype of the *rs3025020 VEGFA* polymorphism

	All subjects	*rs3025020* polymorphism genotype					*p*-Value	
		CC genotype	CT genotype	TT genotype	CC/CT genotype	CT/TT genotype	CC vs. CT/TT	TT vs. CC/CT
**Number**	**844**	**407**	**354**	**83**	**761**	**437**		
Demographic characteristics								
Age y, mean ± SD	50.6 ± 14.4	50.9 ± 14.3	49.9 ± 14.2	51.9 ± 15.3	50.4 ± 14.3	50.3 ± 14.4	0.59	0.38
Sex male, *N* (%)	498<br>(59)	235<br>(57.7)	218<br>(61.6)	45<br>(54.2)	453<br>(59.5)	263<br>(60.2)	OR = 0.90<br>(0.68, 1.18)<br>*p* = 0.47	OR = 0.80 (0.51, 1.26)<br>*p* = 0.35
Clinical and hematologic characteristics								
Hemoglobin g/L, (*N*)<br>mean ± SD	(838)<br>128 ± 29	(406)<br>128 ± 29	(350)<br>128 ± 29	(82)<br>126 ± 26	(756)<br>128 ± 29	(432)<br>128 ± 28	0.95	0.39
White-blood cell count x10E9/L, (*N*) mean ± SD	(832)<br>9.9 ± 6.4	(404)<br>9.5 ± 5.7	(347)<br>10.6 ± 7.2	(81)<br>9.5 ± 5.7	(751)<br>10.0 ± 6.5	(428)<br>10.4 ± 6.9	0.04	0.51
Platelet count x10E9/L, (*N*)<br>mean ± SD	(834)<br>510 ± 347	(402)<br>512 ± 347	(351)<br>507 ± 345	(81)<br>518 ± 356	(753)<br>510 ± 346	(432)<br>509 ± 347	0.92	0.84
Spleen index cm xE2, (*N*)<br>mean ± SD	(831)<br>148 ± 99	(399)<br>143 ± 81	(350)<br>157 ± 116	(82)<br>136 ± 97	(749)<br>150 ± 99	(432)<br>153 ± 113	0.12	0.23
Monocyte count x10E9/L, (*N*)<br>mean ± SD	(453)<br>606 ± 507	(217)<br>626 ± 502	(191)<br>570 ± 482	(45)<br>660 ± 623	(408)<br>560 ± 493	(236)<br>587 ± 511	0.41	0.45
Serum lactate dehydrogenase level x ULN, (*N*) mean ± SD	(496)<br>1.64 ± 1.13	(232)<br>1.72 ± 1.23	(189)<br>1.56 ± 1.03	(48)<br>1.56 ± 1.01	(421)<br>1.64 ± 1.14	(237)<br>1.56 ± 1.03	0.12	0.61
Biological characteristics								
Blood CD34-positive cells x10E6/L, (*N*) mean ± SD	(398)<br>59.5 ± 160	(191)<br>62 ± 177	(164)<br>57 ± 146	(43)<br>57.5 ± 130	(355)<br>59.7 ± 163	(207)<br>57.2 ± 142	0.76	0.93
Serum cholesterol mg/dL, (*N*)<br>mean ± SD	(420)<br>159 ± 42.9	(208)<br>159 ± 43.7	(174)<br>156 ± 42.5	(38)<br>171 ± 39	(382)<br>158 ± 43	(212)<br>159 ± 42.2	0.98	0.06
Accessory mutations (ASXL1/ EZH2)<br>*N.* (%)	45/240<br>(18.7)	21/115<br>(18.2)	22/102<br>(21.6)	2/23<br>(8.7)	43/217<br>(19.8)	24/125<br>(19.2)	OR = 0.94<br>(0.49, 1.81);<br>*p* = 0.85	OR = 0.38 (0.09, 1, 70): *p* = 0.21
Cytogenetic abnormalities<br>*N* (%)	86/290<br>(29.6)	43/150<br>(28.7)	36/118<br>(30.5)	7/22<br>(31.8)	79/268<br>(29.5)	43/140<br>(30.7)	OR = 1.10<br>(0.66, 1.82);<br>*p* = 0.70	OR = 0.98 (0.39, 2.46);<br>*p* = 0.97

 © 2021. The Author(s).

Supplementary Table S2 (*Continued*)

	All subjects	*rs3025020* polymorphism genotype					*p*-Value	
		CC genotype	CT genotype	TT genotype	CC/CT genotype	CT/TT genotype	CC vs. CT/TT	TT vs. CC/CT
Number	844	407	354	83	761	437		
hs-CRP ng/mL, (*N*) mean ± SD	(237)<br>0.72 ± 1.57	(120)<br>0.75 ± 1.7	(90)<br>0.63 ± 1.34	(27)<br>0.91 ± 1.68	(210)<br>0.70 ± 1.55	(117)<br>0.69 ± 1.42	0.77	0.51
sIL-2R ng/mL, (*N*) mean ± SD	(106)<br>1,735 ± 1,410	(52)<br>1,635 ± 1,523	(41)<br>1,881 ± 1,452	(13)<br>1,677 ± 625	(93)<br>1,743 ± 1,489	(54)<br>1,832 ± 1,299	0.47	0.87
Blood CXCR4 expression on CD34-positive cells %, (*N*) mean ± SD	(294)<br>42.2 ± 25	(146)<br>39.5 ± 23.9	(118)<br>42.5 ± 25.6	(30)<br>54.4 ± 25.3	(264)<br>40.8 ± 24.7	(148)<br>44.9 ± 25.9	0.06	**0.005**
BM fibrosis grade 0, *N* (%)	259/842<br>(30.8)	118/406<br>(29.1)	118/353<br>(33.4)	23/83<br>(27.7)	236/759<br>(31.1)	141/436<br>(32.3)	OR = 1.10<br>(0.64, 1.15);<br>$p = 0.30$	OR = 0.85 (0.51, 1.41)<br>$p = 0.53$

Abbreviations: hs-CRP = high-sensitivity C-reactive protein; sIL-2R = soluble IL-2 receptor; ULN, upper limit of normal.

 © 2021. The Author(s).

Supplementary Table S3 Clinical and laboratory features at diagnosis of patients with PMF and *JAK2*^{V617F}-positive mutation stratified by the genotype of the *rs3025020 VEGFA* polymorphism

		rs3025020 polymorphism genotype					*p*-Value	
	All subjects	CC genotype	CT genotype	TT genotype	CC/CT genotype	CT/TT genotype	CC vs. CT/TT	TT vs. CC/CT
Clinical and hematologic characteristics								
Hemoglobin g/L, (*N*); mean ± SD	(535) 134 ± 29	(274) 132 ± 31	(216) 136 ± 28	(45) 130 ± 28	(490) 134 ± 30	(261) 135 ± 28	0.34	0.35
White-blood cell count x10^9/L, (*N*) mean ± SD	(533) 10.6 ± 6.9	(273) 9.8 ± 5.9	(215) 11.7 ± 7.9	(45) 10.7 ± 7.1	(488) 10.6 ± 6.9	(260) 11.5 ± 7.7	**0.005**	0.92
Platelet count x10E9/L, (*N*) mean ± SD	(532) 473 ± 291	(271) 464 ± 292	(216) 485 ± 300	(45) 467 ± 247	(487) 473 ± 295	(261) 482 ± 291	0.49	0.87
Spleen index cm E2, (*N*) mean ± SD	(533) 151 ± 88	(270) 148 ± 83	(217) 158 ± 97	(46) 137 ± 72	(487) 153 ± 89	(263) 154 ± 93	0.42	0.25
Monocyte count x10E9/L, (*N*) mean ± SD	(285) 607 ± 545	(141) 611 ± 517	(122) 576 ± 546	(22) 752 ± 702	(263) 595 ± 530	(144) 602 ± 573	0.92	0.19
Serum lactate dehydrogenase level x ULN, (*N*) mean ± SD	(303) 1.49 ± 1.02	(155) 1.58 ± 1.17	(122) 1.38 ± 0.83	(26) 1.45 ± 0.82	(277) 1.49 ± 1.04	(148) 1.39 ± 0.83	0.10	0.85
Blood CD34- positive cells x10E6/L, (*N*) mean ± SD	(256) 52 ± 160	(130) 63 ± 199	(104) 34 ± 85	(22) 65 ± 165	(234) 50 ± 159	(126) 39 ± 103	0.24	0.68
Serum cholesterol mg/dL, (*N*) mean ± SD	(265) 160 ± 39	(134) 157 ± 42	(111) 155 ± 35	(20) 166 ± 44	(245) 156 ± 39	(131) 157 ± 36	0.94	0.25
Accessory mutations (ASXL1/ EZH2), *N*. (%)	20/148 (13.)%	8/71 (11.3)	12/66 (18.2)	0/11 (0)	20/137 (14.6)	12/77 (15.6)	OR = 0.69 (0.26, 1.79) $p = 0.44$	OR = 0.25 (0.01, 4.39)$p = 0.34$
Chromosomal abnormalities, *N* (%)	60/189 (31.7)	34/101 (33.7)	21/75 (28)	5/13 (38.5)	55/176 (31.2)	26/88 (29.5)	OR = 1.21 (0.65, 2.24) $p = 0.54$	OR = 1.37 (0.43, 4.39) $p = 0.59$
hs-CRP ng/mL, (*N*) mean ± SD	(150) 0.61 ± 1.17	(79) 0.58 ± 0.83	(56) 0.58 ± 1.51	(15) 0.85 ± 1.38	(135) 0.58 ± 1.15	(71) 0.64 ± 1.47	0.75	0.39
sIL-2R ng/mL, (*N*) mean ± SD	(56) 1,856 ± 1,477	(29) 1,929 ± 1,846	(20) 1,747 ± 1,051	(7) 1,866 ± 725	(49) 1,854 ± 1,560	(27) 1,777 ± 965	0.70	0.98
Blood CXCR4 expression on CD34-postive cells %, (*N*) mean ± SD	(186) 43 ± 25	(99) 41 ± 24	(72) 45 ± 25	(15) 50 ± 27	(171) 43 ± 24	(87) 46 ± 25	0.12	0.28
BM fibrosis grade 0, *N* (%)	184/540 (34.1)	90/276 (32.1)	80/218 (36.7)	14/46 (30.4)	170/494 (34.4)	94/264 (35.6)	OR = 0.87 (0.61, 1.25) $p = 0.46$	OR = 0.83 (0.43, 1.60) $p = 0.58$

Abbreviations: hs-CRP, high- sensitivity C-reactive protein; sIL-2R, soluble IL-2 receptor; ULN, upper limit of normal.
Note: Spleen size was measured using the spleen index calculated by multiplying the length of the longitudinal axis by the transverse axis.

 © 2021. The Author(s).

Supplementary Table S4 Clinical and laboratory features at diagnosis of patients with PMF and *JAK2*^{V617F}-negative mutation stratified by the genotype of the *rs3025020 VEGFA* polymorphism

		rs3025020 polymorphism genotype					*p*-Value	
	All subjects	CC genotype	CT genotype	TT genotype	CC/CT genotype	CT/TT genotype	CC vs. CT/TT	TT vs. CC/CT
Clinical and hematologic characteristics								
Hemoglobin g/L, (*N*) mean ± SD	(281)<br>118 ± 25	(124)<br>119 ± 25	(123)<br>117 ± 27	(34)<br>120 ± 23	(247)<br>118 ± 26	(157)<br>117 ± 26	0.56	0.71
White-blood cell count x10E9/L, (*N*) mean ± SD	(280)<br>8.7 ± 5.2	(124)<br>8.9 ± 5.3	(123)<br>8.8 ± 5.6	(33)<br>7.9 ± 2.9	(247)<br>8.8 ± 5.4	(156)<br>8.6 ± 5.2	0.67	0.35
Platelet count x10E9/L, (*N*) mean ± SD	(281)<br>577 ± 425	(124)<br>615 ± 429	(124)<br>540 ± 414	(33)<br>574 ± 458	(248)<br>578 ± 422	(157)<br>547 ± 422	0.18	0.96
Spleen index cmE2, (*N*) mean ± SD	(276)<br>145 ± 120	(121)<br>133 ± 79	(122)<br>159 ± 147	(33)<br>137 ± 129	(243)<br>146 ± 119	(155)<br>155 ± 143	0.13	0.68
Monocyte count x10E9/L, (*N*) mean ± SD	(159)<br>607 ± 445	(72)<br>663 ± 486	(66)<br>556 ± 348	(21)<br>578 ± 561	(138)<br>612 ± 427	(87)<br>561 ± 406	0.15	0.74
Serum lactate dehydrogenase level x ULN, (*N*) mean ± SD	(157)<br>1.88 ± 1.76	(73)<br>1.94 ± 1.06	(65)<br>1.88 ± 1.28	(19)<br>1.66 ± 1.27	(138)<br>1.91 ± 1.16	(84)<br>1.83 ± 1.27	0.56	0.39
Blood CD34. positive cells x10E6/L, (*N*) mean ± SD	(132)<br>76 ± 164	(55)<br>61 ± 123	(57)<br>100 ± 213	(20)<br>52 ± 82	(112)<br>81 ± 175	(77)<br>88 ± 188	0.35	0.47
Serum cholesterol mg/dL, (*N*) mean ± SD	(148)<br>163 ± 49	(72)<br>162 ± 47	(60)<br>159 ± 54	(16)<br>181 ± 32	(132)<br>161 ± 50	(76)<br>164 ± 51	0.78	0.12
Accessory mutations (ASXL1/ EZH2), *N*. (%)	25/87<br>(28.7)	13/42<br>(30.9)	10/35<br>(28.6)	2/10<br>(20)	23/77<br>(29.9)	12/45<br>(16.2)	OR = 1,23 (0.48, 3.12)<br>*p* = 0.60	OR = 0.59 (0.11, 2.98)<br>*p* = 0.52
Chromosomal abnormalities, *N* (%)	25/99<br>(25.2)	8/48<br>(16.7)	15/42<br>(35.7)	2/9<br>(22.2)	23/90<br>(25.5)	17/51<br>(33.3)	OR = 0.40 (0.15, 1.04)<br>*p* = 0.06	OR = 0.83 (0.16, 4.29)<br>*p* = 0.82
hs-CRP ng/mL, (*N*) mean ± SD	(82)<br>0.90 ± 2.1	(38)<br>1.01 ± 2.7	(33)<br>0.72 ± 1.05	(11)<br>1.07 ± 2.1	(41)<br>1.04 ± 2.6	(33)<br>0.72 ± 1.0	0.66	0.77
sIL-2R ng/mL, (*N*) mean ± SD	(47)<br>1,612 ± 1,374	(21)<br>1,254 ± 925	(21)<br>2009 ± 1,770	(5)<br>1,449 ± 500	(42)<br>1,632 ± 1,446	(26)<br>1,901 ± 1,611	0.11	0.78
Blood CXCR4 expression on CD34-positive cells %, (*N*) mean ± SD	(102)<br>41 ± 26	(43)<br>37 ± 23	(45)<br>38 ± 27	(14)<br>60 ± 24	(88)<br>38 ± 25	(59)<br>43 ± 27	0.21	**0.002**
BM fibrosis grade 0, *N* (%)	69/281<br>(24.5)	26/123<br>(21.1)	35/124<br>(28.2)	8/34<br>(23.5)	61/247<br>(24.7)	43/158<br>(27.2)	OR = 0.72 (0.41, 1.25)<br>*p* = 0.24	OR = 0.93 (0.40, 2.18)<br>*p* = 0.88

Abbreviations: hs-CRP, high- sensitivity C-reactive protein; sIL-2R, soluble IL-2 receptor; ULN, upper limit of normal.
Note: Spleen size was measured using the spleen index calculated by multiplying the length of the longitudinal axis by the transverse axis.

 © 2021. The Author(s).

Supplementary Table S5 Clinical and laboratory features at diagnosis of patients with PMF and *JAK2*^{V617F} homozygous mutation stratified by the genotype of the *rs3025020 VEGFA* polymorphism

		rs3025020 polymorphism genotype					*p*-Value	
	**All subjects**	**CC genotype**	**CT genotype**	**TT genotype**	**CC/CT genotype**	**CT/TT genotype**	**CC vs. CT/TT**	**TT vs. CC/CT**
Clinical and hematologic characteristics								
Hemoglobin g/L, (*N*) mean ± SD	(202)<br>139 ± 32	(96)<br>139 ± 34	(86)<br>141 ± 29	(20)<br>130 ± 29	(182)<br>140 ± 32	(106)<br>139 ± 30	0.96	**0.15**
White-blood cell count, x10E9/L, (*N*) mean ± SD	(201)<br>13 ± 7.9	(96)<br>11.3 ± 5.4	(85)<br>15.3 ± 9.3	(20)<br>12.3 ± 9.2	(181)<br>13.2 ± 7.7	(105)<br>14.7 ± 9.3	**0.002**	0.62
Platelet count x10E9/L, (*N*) mean ± SD	(199)<br>441 ± 253	(93)<br>457 ± 282	(86)<br>430 ± 217	(20)<br>413 ± 260	(179)<br>444 ± 253	(106)<br>427 ± 224	0.40	0.61
Spleen index cmE2, (*N*) mean ± SD	(199)<br>180 ± 116	(92)<br>178 ± 110	(86)<br>189 ± 126	(21)<br>156 ± 98	(178)<br>183 ± 117	(107)<br>183 ± 121	0.74	0.31
Monocyte count x10E9/L, (*N*) mean ± SD	(96)<br>713 ± 709	(38)<br>693 ± 665	(48)<br>684 ± 685	(10)<br>926 ± 988	(86)<br>688 ± 672	(58)<br>726 ± 741	0.83	0.32
Serum lactate dehydrogenase level x ULN, (*N*) mean ± SD	(108)<br>1.90 ± 1.21	(45)<br>2.00 ± 1.43	(51)<br>1.81 ± 1.06	(12)<br>1.90 ± 0.99	(96)<br>1.90 ± 1.24	(63)<br>1.83 ± 1.04	0.47	0.99
Blood CD34-positive cells x10E6/L, (*N*) mean ± SD	(67)<br>104 ± 241	(27)<br>145 ± 334	(32)<br>74 ± 138	(8)<br>87 ± 195	(59)<br>106 ± 248	(40)<br>77 ± 148	0.26	0.83
Serum cholesterol mg/dL, (*N*) mean ± SD	(101)<br>143 ± 38	(45)<br>140 ± 40	(47)<br>143 ± 34	(9)<br>153 ± 48	(92)<br>142 ± 38	(56)<br>145 ± 37	0.51	**0.39**
BM fibrosis grade 0, *N* (%)	48/203<br>(23.6)	24/96<br>(25)	22/86<br>(25.6)	2/21<br>(9.5)	46/182<br>(25.3)	24/107<br>(22.4)	OR = 1.15 (0.60, 2.20)<br>$p = 0.67$	OR = 0.31 (0.07, 1.38)<br>$p = 0.12$

Abbreviation: ULN, upper limit of normal.
Note: Spleen size was measured using the spleen index calculated by multiplying the length of the longitudinal axis by the transverse axis.

 © 2021. The Author(s).

Supplementary Table S6 Clinical and laboratory features at diagnosis of patients with PMF and *CALR* mutation stratified by the genotype of the *rs3025020 VEGFA* polymorphism

	All subjects	*rs3025020* polymorphism genotype					*p*-Value	
		CC genotype	CT genotype	TT genotype	CC/CT genotype	CT/TT genotype	CC vs. CT/TT	TT vs. CC/CT
Clinical and hematologic characteristics								
Hemoglobin g/L, (*N*) mean ± SD	(170)<br>124 ± 21	(71)<br>127 ± 20	(78)<br>123 ± 23	(21)<br>122 ± 21	(149)<br>125 ± 22	(99)<br>122 ± 22	0.17	0.56
White-blood cell count x10E9/L, (*N*) mean ± SD	(169)<br>8.4 ± 3.6	(71)<br>8.9 ± 4.2	(78)<br>8.1 ± 3.3	(20)<br>8.1 ± 2.7	(149)<br>8.5 ± 3.8	(98)<br>8.1 ± 3.2	0.13	0.66
Platelet count x10E9/L, (*N*) mean ± SD	(170)<br>693 ± 402	(71)<br>782 ± 419	(79)<br>615 ± 352	(20)<br>685 ± 470	(150)<br>694 ± 393	(99)<br>629 ± 378	**0.013**	0.92
Spleen index cmE2, (*N*) mean ± SD	(165)<br>145 ± 122	(68)<br>131 ± 75	(77)<br>165 ± 160	(20)<br>114 ± 46	(145)<br>149 ± 128	(97)<br>154 ± 146	0.23	0.22
Monocyte count x10E9/L, (*N*) mean ± SD	(100)<br>593 ± 451	(41)<br>673 ± 478	(43)<br>521 ± 319	(16)<br>579 ± 644	(84)<br>595 ± 409	(59)<br>573 ± 426	0.14	0.13
Serum lactate dehydrogenase level x ULN, (*N*) mean ± SD	(94)<br>1.86 ± 1.14	(40)<br>1.82 ± 0.91	(42)<br>1.92 ± 1.22	(12)<br>1.82 ± 1.56	(82)<br>1.87 ± 1.07	(54)<br>1.89 ± 1.29	0.75	0.88
Biological characteristics								
Blood CD34-positive cells x10E6/L. (*N*) mean ± SD	(74)<br>80 ± 157	(27)<br>63 ± 138	(33)<br>101 ± 189	(14)<br>61 ± 97	(60)<br>82 ± 168	(47)<br>90 ± 167	0.49	0.63
Serum cholesterol, mg/dL, (*N*) mean ± SD	(86)<br>168 ± 45	(38)<br>165 ± 45	(37)<br>168 ± 47	(11)<br>180 ± 31	(75)<br>166 ± 46	(48)<br>170 ± 44	0.58	0.34
hs-CRP ng/mL, (*N*) mean ± SD	(43)<br>0.63 ± 1.31	(15)<br>0.17 ± 0.22	(19)<br>0.67 ± 1.08	(9)<br>1.29 ± 2.33	(34)<br>0.45 ± 0.84	(28)<br>0.87 ± 1.57	0.09	0.09
Blood CXCR4 expression on CD34-positve cells %, (*N*) mean ± SD	(58)<br>45 ± 25	(21)<br>38 ± 20	(26)<br>44 ± 27	(11)<br>61 ± 23	(47)<br>41 ± 24	(37)<br>49 ± 27	0.10	**0.016**
BM fibrosis grade 0, *N* (%)	46/170<br>(27)	17/70<br>(24.3)	25/79<br>(31.6)	4/21<br>(19)	42/149<br>(28.2)	29/100<br>(29)	OR = 1.27<br>(0.63, 2.55)*p* = 0.49	OR = 0.59<br>(0.19, 1,88)*p* = 0.38

Abbreviations: hs-CRP, high- sensitivity C-reactive protein; ULN, upper limit of normal.
Note: Spleen size was measured using the spleen index calculated by multiplying the length of the longitudinal axis by the transverse axis.

 © 2021. The Author(s).

Supplementary Table S7 Clinical and laboratory features at diagnosis of patients with PMF and *CALR*-type 1 mutation stratified by the genotype of the *rs3025020 VEGFA* polymorphism

	All subjects	*rs3025020* polymorphism genotype					*p*-Value	
		CC genotype	CT genotype	TT genotype	CC/CT genotype	CT/TT genotype	CC vs. CT/TT	TT vs. CC/CT
Clinical and hematologic characteristics								
Hemoglobin g/L, (*N*) mean ± SD	(122)<br>123 ± 21	(53)<br>125 ± 21	(56)<br>122 ± 22	(13)<br>115 ± 21	(109)<br>124 ± 22	(69)<br>121 ± 22	0.23	0.06
White-blood cell count x10E9/L, (*N*) mean ± SD	(121)<br>8.6 ± 4.1	(53)<br>9.2 ± 4.7	(56)<br>8.0 ± 3.5	(12)<br>8.6 ± 3.2	(109)<br>8.6 ± 4.2	(68)<br>8.1 ± 3.4	(0.18)	0.92
Platelet count x10E9/L, (*N*) mean ± SD	(121)<br>670 ± 396	(53)<br>745 ± 398	(56)<br>602 ± 339	(12)<br>658 ± 586	(109)<br>672 ± 374	(68)<br>612 ± 389	0.07	0.69
Spleen index cmE2, (*N*) mean ± SD	(118)<br>154 ± 130	(51)<br>136 ± 79	(55)<br>175 ± 172	(12)<br>129 ± 56	(106)<br>156 ± 136	(67)<br>167 ± 158	0.20	0.64
Monocyte count x10E9/L, (*N*) mean ± SD	(71)<br>598 ± 474	(31)<br>662 ± 452	(31)<br>514 ± 333	(9)<br>668 ± 856	(62)<br>588 ± 401	(40)<br>548 ± 490	0.49	0.45
Serum lactate dehydrogenase level x ULN, (*N*) mean ± SD	(67)<br>2.00 ± 1.24	(31)<br>1.85 ± 0.94	(29)<br>2.04 ± 1.39	(7)<br>2.54 ± 1.72	(60)<br>1.94 ± 1.17	(36)<br>2.14 ± 1.45	0.34	0.20
Biological characteristics								
Blood CD34-positive cells x10E6/L, (*N*) mean ± SD	(54)<br>72 ± 126	(20)<br>74 ± 159	(26)<br>62 ± 102	(8)<br>97 ± 117	(46)<br>67 ± 128	(34)<br>70 ± 105	0.91	0.54
Serum cholesterol mg/dL, (*N*) mean ± SD	(66)<br>165 ± 44	(29)<br>167 ± 50	(31)<br>163 ± 42	(6)<br>171 ± 31	(60)<br>165 ± 46	(37)<br>164 ± 41	0.84	0.73
Blood CXCR4 expression on CD34-postive cells %, (*N*) mean ± SD	(41)<br>47 ± 27	(15)<br>43 ± 23	(19)<br>47 ± 30	(7)<br>57 ± 27	(34)<br>45 ± 27	(26)<br>50 ± 29	0.29	0.13

Abbreviation: ULN, upper limit of normal.
Note: Spleen size was measured using the spleen index calculated by multiplying the length of the longitudinal axis by the transverse axis.

 © 2021. The Author(s).

Supplementary Table S8 Clinical and laboratory features at diagnosis of patients with PMF and *CALR*-type 2 mutation stratified by the genotype of the *rs3025020 VEGFA* polymorphism

	All subjects	rs3025020 polymorphism genotype					*p*-Value	
		CC genotype	CT genotype	TT genotype	CC/CT genotype	CT/TT genotype	CC vs. CT/TT	TT vs. CC/CT
Clinical and hematologic characteristics								
Hemoglobin g/L, (*N*) mean ± SD	(44)<br>127 ± 22	(15)<br>131 ± 19	(21)<br>122 ± 26	(8)<br>133 ± 14	(36)<br>126 ± 23	(29)<br>125 ± 24	0.46	0.55
White-blood cell count x10E9/L, (*N*) mean ± SD	(44)<br>8.1 ± 2.3	(15)<br>8.4 ± 1.3	(21)<br>8.1 ± 3.0	(8)<br>7.4 ± 1.3	(36)<br>8.2 ± 2.4	(29)<br>7.9 ± 2.6	0.26	0.36
Platelet count, x10E9/L, (*N*) mean ± SD	(45)<br>742 ± 395	(15)<br>966 ± 480	(22)<br>595 ± 309	(8)<br>726 ± 240	(37)<br>745 ± 424	(30)<br>630 ± 295	**0.003**	0.89
Spleen index cmE2, (*N*) mean ± SD	(43)<br>125 ± 99	(14)<br>119 ± 69	(21)<br>142 ± 130	(8)<br>91 ± 3.5	(35)<br>133 ± 109	(29)<br>128 ± 112	0.79	0.30

Note: Spleen size was measured using the spleen index calculated by multiplying the length of the longitudinal axis by the transverse axis.

Supplementary Table S9 Clinical and laboratory features at diagnosis of patients with PMF and *MPL* mutation stratified by the genotype of the *rs3025020 VEGFA* polymorphism

	All subjects	rs3025020 polymorphism genotype					*p*-Value	
		CC genotype	CT genotype	TT genotype	CC/CT genotype	CT/TT genotype	CC vs. CT/TT	TT vs. CC/CT
Clinical and hematologic characteristics								
Hemoglobin g/L, (*N*) mean ± SD	(44)<br>109 ± 23	(24)<br>111 ± 20	(11)<br>108 ± 28	(9)<br>108 ± 23	(35)<br>110 ± 23	(20)<br>108 ± 25	0.66	0.84
White-blood cell count x10E9/L, (*N*) mean ± SD	(44)<br>7.8 ± 4.1	(24)<br>7.3 ± 3.8	(11)<br>10.1 ± 5.1	(9)<br>6.2 ± 1.9	(35)<br>8.2 ± 4.4	(20)<br>8.4 ± 4.4	0.39	0.19
Platelets x10E9/L, (*N*) mean ± SD	(44)<br>445 ± 289	(24)<br>451 ± 302	(11)<br>539 ± 263	(9)<br>316 ± 259	(35)<br>479 ± 290	(20)<br>439 ± 279	0.89	0.13
Spleen index cmE2, (*N*) mean ± SD	(44)<br>157 ± 137	(24)<br>147 ± 111	(11)<br>137 ± 83	(9)<br>207 ± 232	(35)<br>143 ± 102	(20)<br>168 ± 166	0.62	0.22

Abbreviations: hs-CRP, high-sensitivity C-reactive protein; sIL-2R, soluble IL-2 receptor; ULN, upper limit of normal.
Note: Spleen size was measured using the spleen index calculated by multiplying the length of the longitudinal axis by the transverse axis.

 © 2021. The Author(s).

Supplementary Table S10 Clinical and laboratory features at diagnosis of patients with PMF and *triple negative* mutation stratified by the genotype of the *rs3025020 VEGFA* polymorphism

	All subjects	rs3025020 polymorphism genotype					*p*-Value	
		CC genotype	CT genotype	TT genotype	CC/CT genotype	CT/TT genotype	CC vs. CT/TT	TT vs. CC/CT
Clinical and hematologic characteristics								
Hemoglobin g/L, (*N*) mean ± SD	(67)<br>108 ± 31	(29)<br>107 ± 31	(34)<br>106 ± 30	(4)<br>134 ± 24	(63)<br>106 ± 30	(38)<br>109 ± 31	0.74	0.08
White-blood cell count x10E9/L, (*N*) mean ± SD	(67)<br>10 ± 8.2	(29)<br>9.9 ± 7.9	(34)<br>9.9 ± 8.9	(4)<br>10.9 ± 4.3	(63)<br>9.9 ± 8.4	(38)<br>10 ± 8.5	0.96	0.81
Platelets x10E9/L, (*N*) mean ± SD	(67)<br>369 ± 458	(29)<br>339 ± 345	(34)<br>367 ± 527	(4)<br>593 ± 612	(63)<br>355 ± 449	(38)<br>391 ± 532	0.65	0.31
Spleen index cmE2, (*N*) mean ± SD	(67)<br>138 ± 103	(29)<br>124 ± 56	(34)<br>155 ± 134	(4)<br>97 ± 15	(63)<br>141 ± 105	(38)<br>149 ± 127	0.34	0.42

Note: Spleen size was measured using the spleen index calculated by multiplying the length of the longitudinal axis by the transverse axis.

 © 2021. The Author(s).
